# Supplementary material for: Proximity labeling proteomics reveals critical regulators for inner nuclear membrane protein degradation in plants
Source: Nat Commun. 2020 Jun 29;11:3284. doi: 10.1038/s41467-020-16744-1 (PMC7324386; doi:10.1038/s41467-020-16744-1)
Supplement: Supplementary file 2 — Supplementary Information [file 41467_2020_16744_MOESM2_ESM.pdf]

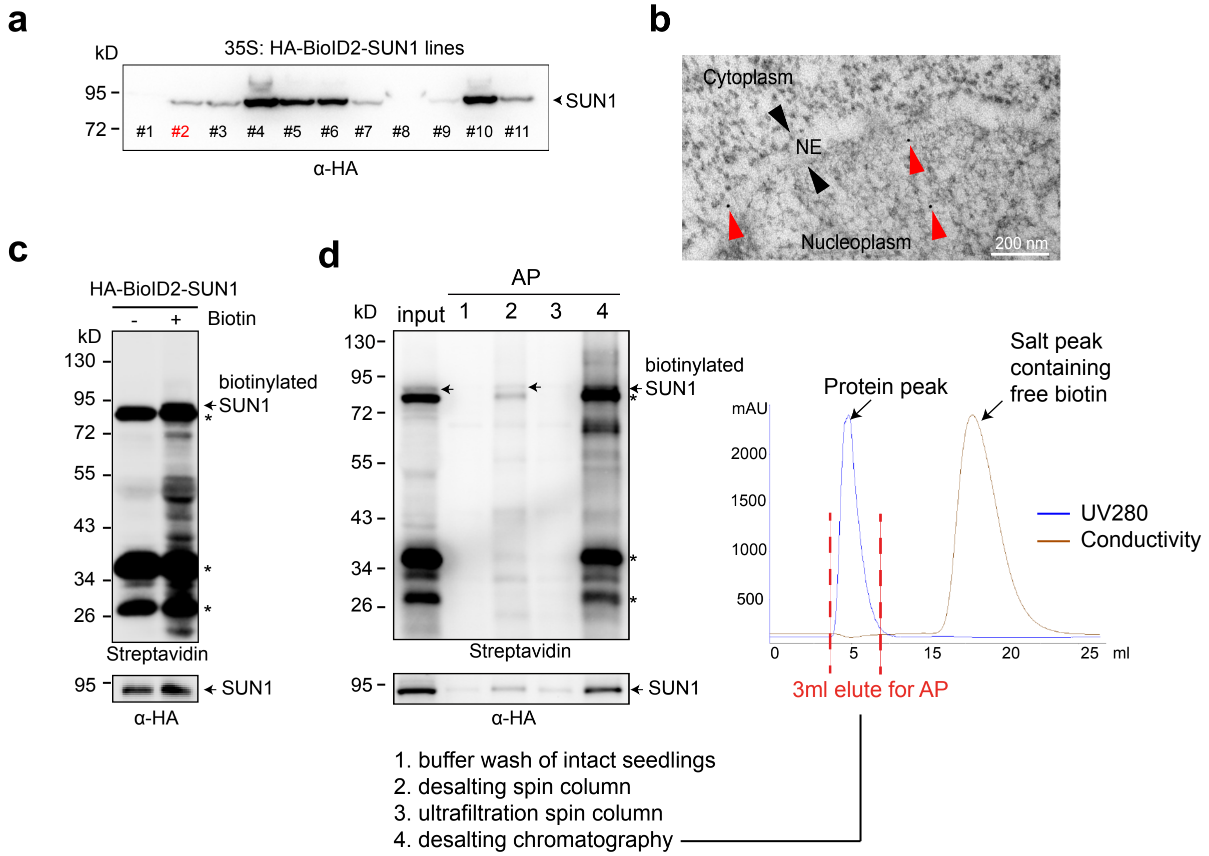

Supplementary Fig. 1 Further validation of the *35S: HA-BioID2-SUN1* transgenic line and optimization of free biotin depletion for PL-LFQMS. a, The expression level of HA-BioID2-SUN1 in the transgenic line used for the previously reported PL-LFQMS profiling is relatively low among other lines. **b**, Further validation of the nuclear envelope (NE) localization of SUN1 in root cells of *35S: HA-BioID2-SUN1* plants by transmission immunoelectron microscopy. The NE is marked by black arrowheads. Golden particles are indicated by red arrowheads. **c**, Futher validation of the inducible biotinylation in *35S: HA-BioID2-SUN1* plants. Streptavidin and anti-HA immunoblots of total protein extract from *35S: HA-BioID2-SUN1* Arabidopsis plants with (+) or without (-) 50 μM free biotin treatment. Arrows indicate the HA-BioID2-SUN1 proteins. Asterisks mark naturally biotinylated proteins in Arabidopsis. **d**, Optimization of free biotin depletion for affinity purification (AP) of total biotinylated proteins during PL-LFQMS. Total protein extract (input) from biotin-treated *35S: HA-BioID2-SUN1* Arabidopsis seedlings were directly used for AP (1) or subject to three different methods of free biotin removal before AP, including desalting spin column (2), ultrafiltration spin column (3), and desalting chromatography that was performed on AKTA FPLC protein purification system (4).

a

ID	Name	*p*_ adj_SUN1/WIT1	Log2SUN1/WIT1	*p*_ adj_SUN1/NT	Log2SUN1/NT
AT5G04990	SUN1	6.8E-14	5.21	1.4E-12	4.69
AT4G38930	UFD1C	6.8E-14	5.65	6.6E-09	5.29
AT2G21270	UFD1B	6.8E-14	5.51	8.8E-14	5.1
AT5G52240	PNET7	6.8E-14	5.08	2.4E-06	4.51
AT4G04210	PUX4	6.8E-14	5.13	2.4E-08	4.44
AT1G68790	CRWN3	6.8E-14	4.41	2.5E-03	2.93
AT5G48810	PNET10	5.2E-11	4.74	7.0E-04	3.96
AT4G31430	KAKU4	1.2E-10	3.07	4.7E-02	1.88
AT5G65770	CRWN4	2.1E-10	3.39	1.8E-07	3.99
AT1G67230	CRWN1	6.7E-10	4.1	3.7E-08	5.14
AT1G13220	CRWN2	5.6E-09	3.13	2.4E-02	2.25
AT4G15410	PUX5	4.1E-07	2.88	3.9E-03	2.66
AT5G46070		1.1E-06	2.84	3.7E-04	3.13
AT3G03100		7.8E-04	4.03	3.9E-02	4.08
AT1G04850		1.6E-02	1.46	7.3E-11	4.07
AT3G19390		4.3E-02	1.48	3.3E-02	2.11

b

ID	Name	*p*_ adj_WIT1/SUN1	Log2WIT1/SUN1	*p*_ adj_WIT1/NT	Log2WIT1/NT
AT5G11390	WIT1	6.8E-14	6.88	3.6E-08	6.63
AT5G19320	RanGAP2	6.8E-14	8.24	1.3E-13	7.71
AT3G63130	RanGAP1	4.2E-13	5.4	3.0E-08	6.01
AT3G13360	WIP3	7.6E-13	3.67	7.2E-03	2.52
AT4G26455	WIP1	1.4E-09	3.03	2.5E-04	2.92

c

Name/ID	PUX4 At4G04210	PUX5 At4G15410	UFD1B AT2G21270	UFD1C AT4G38930
*p*_SUN1/NT	4.8E-06	9.1E-05	1.4E-07	1.4E-08
Log2SUN1/NT	4.9	3.57	6.2	6.51
*p*_SUN1/YFP(-)	2.4E-04	3.5E-03	6.7E-06	1.1E-05
Log2SUN1/YFP(-)	3.61	2.47	4.85	4.31
*p*_NEAP1/NT	7.2E-06	2.3E-05	1.2E-04	8.3E-07
Log2NEAP1/NT	4.77	3.99	3.9	5.12
*p*_NEAP1/YFP(-)	3.6E-04	9.1E-04	6.2E-03	9.9E-04
Log2NEAP1/YFP(-)	3.48	2.88	2.55	2.93
*p*_NEMPA/NT	9.8E-05	0.59	0.12	0.06
Log2NEMPA/NT	3.91	0.41	1.36	1.56
*p*_NEMPA/YFP(-)	4.7E-03	0.38	0.98	0.42
Log2NEMPA/YFP(-)	2.62	-0.69	0.02	-0.64
*p*_WIT1/NT	0.43	0.56	0.47	0.50
Log2WIT1/NT	-0.68	0.45	0.62	0.53
*p*_WIT1/YFP(-)	0.03	0.40	0.40	0.04
Log2WIT1/YFP(-)	-1.97	-0.65	-0.72	-1.66
*p*_WIP1/NT	0.06	0.29	0.14	0.01
Log2WIP1/NT	1.69	0.83	1.29	2.30
*p*_WIP1/YFP(-)	0.64	0.73	0.95	0.89
Log2WIP1/YFP(-)	0.40	-0.27	-0.06	0.11
*p*_Nup82/NT	0.62	1.00	0.67	0.30
Log2Nup82/NT	0.43	0.09	-0.37	0.83
*p*_Nup82/YFP(-)	0.31	0.16	0.06	0.09
Log2Nup82/YFP(-)	-0.86	-1.10	-1.71	-1.36
*p*_Nup93a/NT	0.82	0.91	0.65	0.85
Log2Nup93a/NT	-0.19	0.09	-0.39	-0.15
*p*_Nup93a/YFP(-)	0.09	0.20	0.05	0.01
Log2Nup93a/YFP(-)	-1.48	-1.02	-1.74	-2.34
*p*_RHD3/NT	0.42	0.30	0.22	0.19
Log2RHD3/NT	-0.68	-0.81	-1.08	1.06
*p*_RHD3/YFP(-)	0.03	0.02	0.01	0.16
Log2RHD3/YFP(-)	-1.97	-1.91	-2.42	-1.13

Supplementary Fig. 2 Statistics of significantly enriched candidates identified by ratiometric analysis using PL-LFQMS data. a,**b**, Ratiometric analysis statistics for HA-BioID2-SUN1 enriched preys (a) and HA-BioID2-WIT1 enriched preys (b) shown in Fig. 1b. **c**, Ratiometric analysis statistics for PUX4, PUX5, UFD1B, and UFD1C in PL-LFQMS experiments using baits indicated in the first column. Peptide intensity values from LFQMS were used for the analyses. Biotin-treated WT non-transgenic plants (NT) and YFP-BioID2-HA plants without (-) biotin treatment were served as controls. *p*-values were obtained using linear model F-tests.

a

	SUN1/Ctrl1		SUN1/Ctrl2		SUN1(MG132)/Ctrl1		SUN1(MG132)/Ctrl2	
	*p*_value	Log2Ratio	*p*_value	Log2Ratio	*p*_value	Log2Ratio	*p*_value	Log2Ratio
PUX3	0.35	0.85	0.62	-0.44	7.1E-05	6.23	4.5E-04	4.94
PUX4	1.5E-06	5.78	7.6E-05	3.71	7.4E-07	6.95	1.9E-05	4.88
PUX5	3.4E-05	3.40	5.7E-04	2.40	2.1E-06	5.18	1.5E-05	4.17
UFD1B	3.4E-07	6.16	1.1E-05	4.25	1.7E-07	7.38	2.9E-06	5.48
UFD1C	5.6E-06	5.78	5.1E-05	4.49	2.1E-06	7.20	1.2E-05	5.92
NPL4A	0.08	1.54	0.31	-0.85	4.0E-04	4.58	0.03	2.20
CDC48B	0.98	-0.02	0.49	-0.48	1.4E-05	5.82	2.9E-05	5.36
CDC48C	0.01	1.89	0.99	0.01	7.6E-07	7.47	1.1E-05	5.58

b

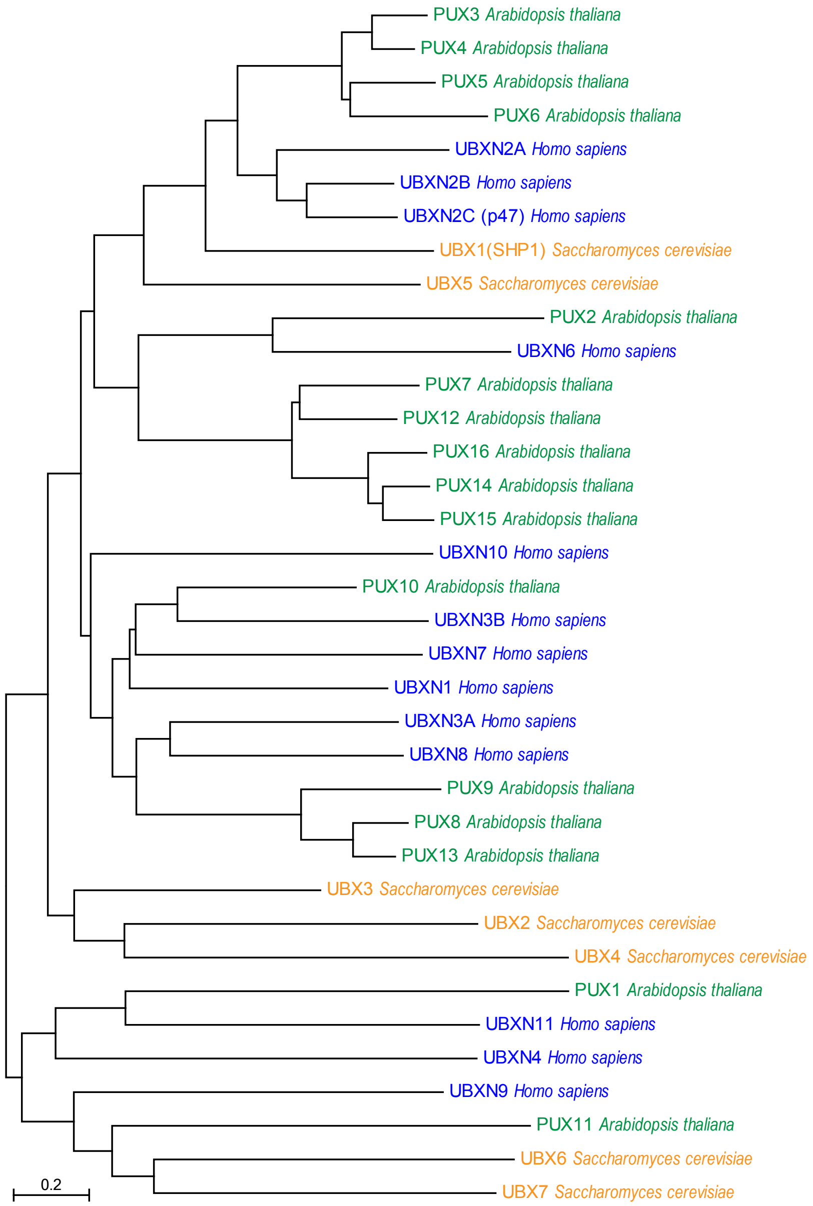

Supplementary Fig. 3 SUN1 probed the entire CDC48 complex and PUX3/4/5 in the presence of MG132. **a**, Ratiometric analysis statistics for PUX3/4/5 and core components of the CDC48 complex in PL-LFQMS experiments using *35S: HA-BioID2-SUN1* plants with or without MG132 treatment. Peptide intensity values from LFQMS were used for the analyses. *p*-values were obtained using linear model F-tests. **b**, Phylogenetic analysis of the full-length protein sequence of UBX domain-containing proteins in Arabidopsis (*Arabidopsis thaliana*, green), yeast (*Saccharomyces cerevisiae*, yellow), and human (*Homo sapiens*, blue). The neighbor-joining tree was generated using MEGA 6.0. Bar corresponds to substitution per site.

a

Name/ID	PUX1 At3G27310	PUX2 At2G01650	PUX3 At4G22150	PUX4 At4G04210	PUX5 At4G15410	PUX6 At3G21660	PUX7 At1G14570	PUX8 At4G11740	PUX9 At4G00752	PUX10 At4G10790	PUX11 At2G43210	PUX12 At3G23605	PUX13 At4G23040	PUX14 At4G14250	PUX15 At1G59550	PUX16 At4G14245
*p*_SUN1/NT	NA	0.10	0.52	4.8E-06	9.1E-05	NA	NA	NA	NA	NA	NA	NA	NA	NA	NA	NA
Log$_2$SUN1/NT	NA	1.98	0.60	4.90	3.57	NA	NA	NA	NA	NA	NA	NA	NA	NA	NA	NA
*p*_SUN1/YFP(-)	NA	0.90	0.98	2.4E-04	3.5E-03	NA	NA	NA	NA	NA	NA	NA	NA	NA	NA	NA
Log$_2$SUN1/YFP(-)	NA	-0.14	-0.03	3.61	2.47	NA	NA	NA	NA	NA	NA	NA	NA	NA	NA	NA
*p*_NEAP1/NT	NA	0.08	2.6E-06	7.2E-06	2.3E-05	NA	NA	NA	NA	NA	NA	NA	NA	NA	NA	NA
Log$_2$NEAP1/NT	NA	2.13	5.57	4.77	3.99	NA	NA	NA	NA	NA	NA	NA	NA	NA	NA	NA
*p*_NEAP1/YFP(-)	NA	1.00	1.5E-05	3.6E-04	9.1E-04	NA	NA	NA	NA	NA	NA	NA	NA	NA	NA	NA
Log$_2$NEAP1/YFP(-)	NA	0.04	4.94	3.48	2.88	NA	NA	NA	NA	NA	NA	NA	NA	NA	NA	NA
*p*_NEMPA/NT	NA	0.2	0.09	9.8E-05	0.59	NA	NA	NA	NA	NA	NA	NA	NA	NA	NA	NA
Log$_2$NEMPA/NT	NA	4.31	1.60	3.91	0.41	NA	NA	NA	NA	NA	NA	NA	NA	NA	NA	NA
*p*_NEMPA/YFP(-)	NA	0.07	0.30	4.7E-03	0.38	NA	NA	NA	NA	NA	NA	NA	NA	NA	NA	NA
Log$_2$NEMPA/YFP(-)	NA	2.19	0.97	2.62	-0.69	NA	NA	NA	NA	NA	NA	NA	NA	NA	NA	NA

b

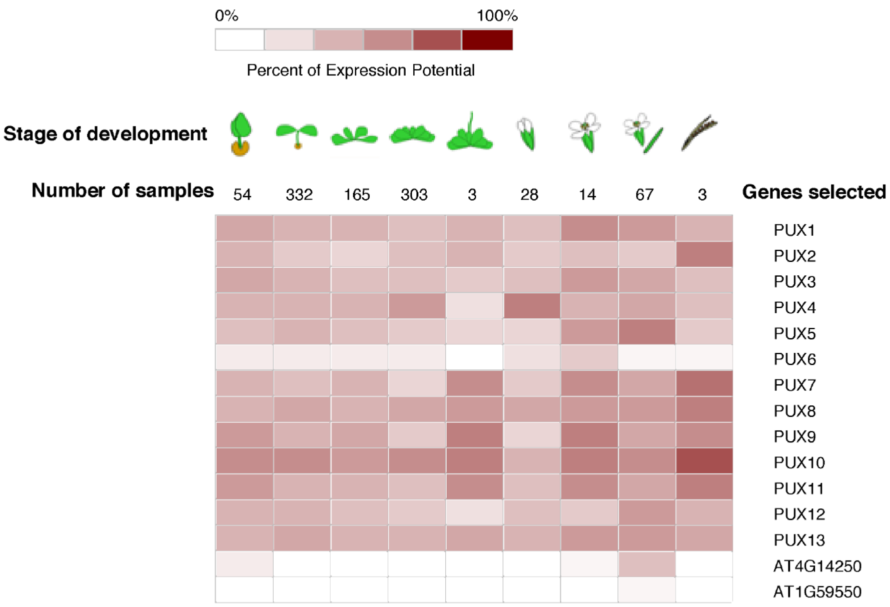

c

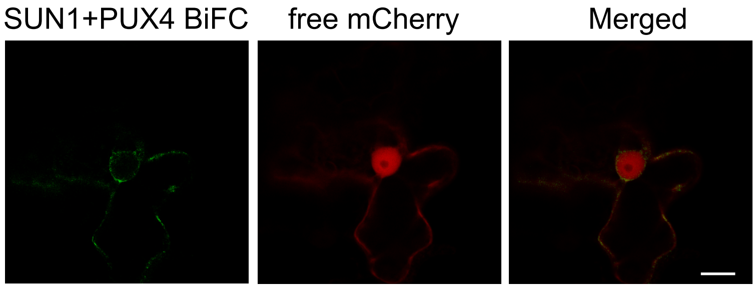

Supplementary Fig. 4 The PUX3/4/5 subgroup selectively associates with INM proteins. a, Ratiometric analysis statistics for 16 AtPUX proteins in PL-LFQMS experiments using INM bait proteins indicated in the first column. Peptide intensity values from LFQMS were used for the analyses. NA represents no detectable signal. Biotin-treated WT non-transgenic plants (NT) and YFP-BioID2-HA plants without (-) biotin treatment were served as controls. *p*-values were obtained using linear model F-tests. **b**, The transcriptional level of At*PUX* genes at different stages of development. Data were obtained from the GENEVESTIGATOR. **c**, The Bimolecular Fluorescence Complementation (BiFC) assay between SUN1 and PUX4. SUN1-nYFP, PUX4-cYFP, and free mCherry were coexpressed transiently in *N. benthamiana*. Leaf epidermal cell was imaged. Bar = 10 μm.

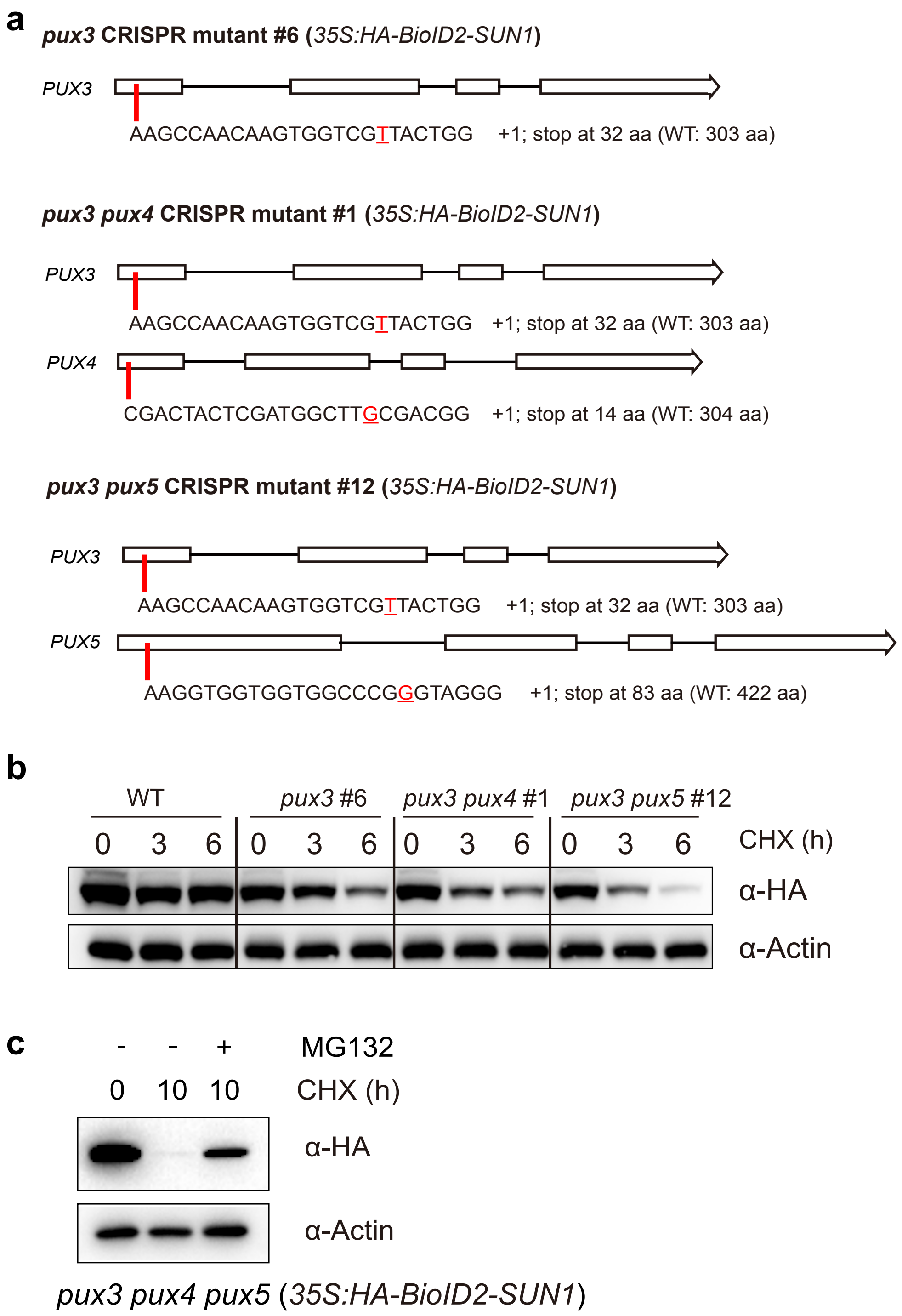

Supplementary Fig. 5 PUX3, PUX4, and PUX5 redundantly protect SUN1 from degradation. a, The *pux3*, *pux3 pux4*, and *pux3 pux5* mutations generated by CRISPR/Cas9 in the *35S: HA-BioID2-SUN1* background. **b**, In vivo degradation assay for HA-BioID2-SUN1 in WT, *pux3*, *pux3 pux4*, and *pux3 pux5* background. Ten-day-old *35S: HA-BioID2-SUN1* seedlings in WT and pux mutant backgrounds were treated with 100 μM CHX for 0, 3, and 6 hrs before total protein was extracted and immunoblotted with anti-HA and anti-actin antibodies. **c**, *In vivo* degradation of SUN1 in *pux3 pux4 pux5* triple mutant was largely compromised by MG132.

Supplementary Note 1

Further validation of the *35S: HA-BioID2-SUN1* transgenic line

Before using the previously published BioID-SUN1 PL-LFQMS data[1] for reanalysis, we performed additional validation on the relative protein expression level, NE localization, and inducible biotinylation of HA-BioID2-SUN1 in the *35S: HA-BioID2-SUN1* transgenic line to further support the specificity and efficiency of our previous profiling. By comparing with other *35S: HA-BioID2-SUN1* lines obtained, we confirmed that the line we used for PL-LFQMS profiling has a relatively low level of HA-BioID2-SUN1 expression (Supplementary Fig. 1a). We also investigated the *in vivo* localization of HA-BioID2-SUN1 in the transgenic line using anti-HA immunogold labeling followed by transmission electron microscopy. We found that HA-BioID2-SUN1 was predominantly associated with the NE and occasionally observed in the nucleoplasm in root cells of the transgenic plants (Supplementary Fig. 1b). However, we did not detect SUN1 in membrane compartments other than the NE. These data support that the BioID2-SUN1-medaited proximity labeling might occurr specifically at the INM. In addition, we treated the transgenic seedlings with 50 μM biotin for 16 hours. Biotin treatment induced significant biotinylation of proteins, including the bait protein itself, suggestive of an efficient and inducible labeling of SUN1 protein and its proximal proteins by BioID2 (Supplementary Fig. 1c).

Optimization of free biotin depletion for PL-LFQMS

The collection of total biotinylated proteins by affinity purification (AP) requires pre-removal of excessive free biotin. However, different from protocols established for cultured mammalian cells and plant protoplasts[2-4], we found that buffer-washing of intact plant tissues was not sufficient to remove free biotin and resulted in failed AP (Supplementary Fig. 1d). Pre-filtering protein extract using desalting or ultrafiltration spin columns improved the AP efficiency but to a limited extent under our buffer conditions. We then performed desalting chromatography, which separates the total protein from the salt fraction that contains free biotin. Protein samples cleaned by desalting chromatography significantly improved the subsequent AP efficiency and yielded sufficient total biotinylated protein for MS identification (Supplementary Fig. 1d). Purified biotinylated proteins were then subject to LFQMS with three biological replicates.

Supplementary References

1 Tang, Y., Huang, A. & Gu, Y. Global profiling of plant nuclear membrane proteome reveals a novel nuclear pore complex component. Nature Plants (2020).

2 Lin, Q. et al. Screening of Proximal and Interacting Proteins in Rice Protoplasts by Proximity-Dependent Biotinylation. Front Plant Sci 8, 749, doi:10.3389/fpls.2017.00749 (2017).

3 Uezu, A. et al. Identification of an elaborate complex mediating postsynaptic inhibition. Science 353, 1123-1129, doi:10.1126/science.aag0821 (2016).

4 Kim, D. I. et al. Probing nuclear pore complex architecture with proximity-dependent biotinylation. Proc Natl Acad Sci U S A 111, E2453-2461, doi:10.1073/pnas.1406459111 (2014).
