# Supplementary material for: Proximity labeling proteomics reveals critical regulators for inner nuclear membrane protein degradation in plants
Source: Nat Commun. 2020 Jun 29;11:3284. doi: 10.1038/s41467-020-16744-1 (PMC7324386; doi:10.1038/s41467-020-16744-1)
Supplement: Supplementary file 3 — Description of Additional Supplementary Files [file 41467_2020_16744_MOESM3_ESM.docx]

**Description of Additional Supplementary Files**

**File name:** Supplementary Data 1

**Description:** Primers used for cloning in this study.

**File name:** Supplementary Data 2

**Description:** The mass spectrometry datasets deposited into ProteomeXchange Consortium.

**File name:** Supplementary Data 3

**Description:** Differential analysis and normalized MS data for BioID2-SUN1, BioID2-WIT1, and PUX5-BioID2 proximity labeling proteomics.
