# Supplementary material for: Proximity labeling proteomics reveals critical regulators for inner nuclear membrane protein degradation in plants
Source: Nat Commun. 2020 Jun 29;11:3284. doi: 10.1038/s41467-020-16744-1 (PMC7324386; doi:10.1038/s41467-020-16744-1)
Supplement: Supplementary file 7 — Reporting Summary [file 41467_2020_16744_MOESM7_ESM.pdf]

nature research

Corresponding author(s): Yangnan Gu

Last updated by author(s): May 8, 2020

Reporting Summary

Nature Research wishes to improve the reproducibility of the work that we publish. This form provides structure for consistency and transparency in reporting. For further information on Nature Research policies, see Authors & Referees and the Editorial Policy Checklist .

Statistics

For all statistical analyses, confirm that the following items are present in the figure legend, table legend, main text, or Methods section.

n/a	Confirmed	
☐	☒	The exact sample size (*n*) for each experimental group/condition, given as a discrete number and unit of measurement
☐	☒	A statement on whether measurements were taken from distinct samples or whether the same sample was measured repeatedly
☐	☒	The statistical test(s) used AND whether they are one- or two-sided<br>*Only common tests should be described solely by name; describe more complex techniques in the Methods section.*
☒	☐	A description of all covariates tested
☐	☒	A description of any assumptions or corrections, such as tests of normality and adjustment for multiple comparisons
☐	☒	A full description of the statistical parameters including central tendency (e.g. means) or other basic estimates (e.g. regression coefficient) AND variation (e.g. standard deviation) or associated estimates of uncertainty (e.g. confidence intervals)
☐	☒	For null hypothesis testing, the test statistic (e.g. *F*, *t*, *r*) with confidence intervals, effect sizes, degrees of freedom and *P* value noted<br>*Give P values as exact values whenever suitable.*
☒	☐	For Bayesian analysis, information on the choice of priors and Markov chain Monte Carlo settings
☒	☐	For hierarchical and complex designs, identification of the appropriate level for tests and full reporting of outcomes
☒	☐	Estimates of effect sizes (e.g. Cohen's *d*, Pearson's *r*), indicating how they were calculated

Our web collection on statistics for biologists contains articles on many of the points above.

Software and code

Policy information about availability of computer code

Data collection	Proteome Discoverer (Version 2.2) developed by Thermo Scientific was used in LCMS workflow for peptide identification.
Data analysis	RStudio (version 1.1.463) was used for statistical analysis and graphics. R is open source and a free software environment. DEP package (version 1.8.0) in R were used for proteomic analyses. MEGA (Version 6.0) software was used for protein sequence analysis from different species and is publicly available.

For manuscripts utilizing custom algorithms or software that are central to the research but not yet described in published literature, software must be made available to editors/reviewers. We strongly encourage code deposition in a community repository (e.g. GitHub). See the Nature Research guidelines for submitting code & software for further information.

Data

Policy information about availability of data

All manuscripts must include a data availability statement. This statement should provide the following information, where applicable:

- Accession codes, unique identifiers, or web links for publicly available datasets
- A list of figures that have associated raw data
- A description of any restrictions on data availability

All the mass spectrometry data generated in this study have been deposited to the ProteomeXchange Consortium via the PRIDE partner repository (Identifier: PXD015920). MS data set names and figures associated with each dataset are listed in Supplementary Table 2. The deposited data are publicly available.

Field-specific reporting

Please select the one below that is the best fit for your research. If you are not sure, read the appropriate sections before making your selection.

Life sciences study design

All studies must disclose on these points even when the disclosure is negative.

Sample size	Due to the large amount of mass spectrometry experiments performed in this study, it is not possible to follow the sample size suggested by a typical Power Analysis. Nevertheless, for each MS profiling of each sample (genotype/treatment), we typically used three replicates, which is more than what is typically used in MS.
Data exclusions	No data were excluded from the analyses.
Replication	All immunoblot data, fluorescent imaging, and yesat-two-hybrid data were successfully replicated and representative results were shown. The mass spectrometry experiments were analysed by incorporating data from two or three replicates to obtain statistical inference.
Randomization	For proximity-labeling proteomic experiments, samples were grouped according to genotypes and treatments. Each sample consists of 0.4 g randomly selected and pooled Arabidopsis seedlings of certain genotype (containing different transgenes) with particular treatment (+/- 50 uM biotin). For fluorescent microscopy, cells expressing target proteins were randomly selected for imaging.
Blinding	This study did not involve paired tests, and blinding is not relevant.

Reporting for specific materials, systems and methods

We require information from authors about some types of materials, experimental systems and methods used in many studies. Here, indicate whether each material, system or method listed is relevant to your study. If you are not sure if a list item applies to your research, read the appropriate section before selecting a response.

Materials & experimental systems

n/a	Involved in the study	
☐	☒	Antibodies
☒	☐	Eukaryotic cell lines
☒	☐	Palaeontology
☐	☒	Animals and other organisms
☒	☐	Human research participants
☒	☐	Clinical data

Methods

n/a	Involved in the study	
☒	☐	ChIP-seq
☒	☐	Flow cytometry
☒	☐	MRI-based neuroimaging

Antibodies

Antibodies used	Anti-HA (3F10, Roche, Cat#11867431001), Anti-Actin (Abiocode, Cat#R3772-1P), Anti-GFP (Takara, Cat#632375), Anti-Ubiquitin (D9D5, Cell Signaling Technology Cat#8081S)
Validation	Anti-HA (3F10 is a mouse monoclonal antibody that recognizes the HA peptide sequence (YPYDVPDYA), derived from influenza hemagglutinin protein. The antibody recognizes its antigenic determinant even when the HA peptide epitope is introduced into unrelated recombinant proteins by a technique known as "epitope tagging".) We used 1:5,000 for western blot and 1:10 for immuno-electron microscopy.<br>Anti-Actin (R3772-1P is a rabbit polyclonal antibody to a fragment of the N-terminal region of Arabidopsis Actin-1 protein and is suitable for western blot.) We used 1:3,000 for western blot.<br>Anti-GFP (Living Colors is a monoclonal antibody produced by hybridoma cells against recombinant full-length Aequorea victoria green fluorescent protein rGFP. This antibody recognizes wild-type GFP, GFPuv, EGFP, destabilized EGFP variants, EBFP, EYFP, ECFP and both N- and C-terminal fusion proteins containing these GFP variants.) We used 1:3,000 for western blot.<br>Anti-Ubiquitin (D9D5#8081 is a monoclonal antibody produced by immunizing animals with a synthetic peptide corresponding to residues surrounding the Lys48 branch of the human diubiquitin chain. K48-linkage Specific Polyubiquitin (D9D5C6) Rabbit mAb detects polyubiquitin chains formed by Lys48 residue linkage. This antibody does not react with monoubiquitin or polyubiquitin chains formed by specific linkage to a different lysine residue.) We used 1:1,000 for western blot.

Animals and other organisms

Policy information about studies involving animals; ARRIVE guidelines recommended for reporting animal research

Laboratory animals	This study did not involve laboratory animals.
Wild animals	This study did not involve wild animals.
Field-collected samples	This study did not involve samples collected from the field.
Ethics oversight	No ethical approval or guidance is required for this study, which used Arabidopsis thaliana as the model organism.

Note that full information on the approval of the study protocol must also be provided in the manuscript.
